# Computational Insight of Phase Transformation and Drug Release Behaviour of Doxycycline-Loaded Ibuprofen-Based In-Situ Forming Gel

**DOI:** 10.3390/pharmaceutics15092315

**Published:** 2023-09-13

**Authors:** Napaphol Puyathorn, Poomipat Tamdee, Jitnapa Sirirak, Siriporn Okonogi, Thawatchai Phaechamud, Takron Chantadee

**Affiliations:** 1 Programme of Pharmaceutical Technology, Faculty of Pharmacy, Silpakorn University, Nakhon Pathom 73000, Thailand; puyathorn_n@old.silpakorn.edu; 2 Department of Chemistry, Faculty of Science, Silpakorn University, Nakhon Pathom 73000, Thailand; 3 Natural Bioactive and Material for Health Promotion and Drug Delivery System Group (NBM Group), Faculty of Pharmacy, Silpakorn University, Nakhon Pathom 73000, Thailand; 4 Department of Pharmaceutical Sciences, Faculty of Pharmacy, Chiang Mai University, Chiang Mai 50200, Thailand; 5 Center of Excellence in Pharmaceutical Nanotechnology, Chiang Mai University, Chiang Mai 50200, Thailand; 6 Department of Industrial Pharmacy, Faculty of Pharmacy, Silpakorn University, Nakhon Pathom 73000, Thailand

**Keywords:** doxycycline, ibuprofen, dimethyl sulfoxide, *N*-methyl-2-pyrrolidone, phase transformation, in-situ forming gels, molecular dynamics simulation

## Abstract

This research investigates the gel formation behaviour and drug-controlling performance of doxycycline-loaded ibuprofen-based in-situ forming gels (DH-loaded IBU-based ISGs) for potential applications in periodontal treatment. The investigation begins by exploring the physical properties and gel formation behaviour of the ISGs, with a particular focus on determining their sustained release capabilities. To gain a deeper understanding of the molecular interactions and dynamics within the ISGs, molecular dynamic (MD) simulations are employed. The effects of adding IBU and DH on reducing surface tension and water tolerance properties, thus affecting molecular properties. The phase transformation phenomenon is observed around the interface, where droplets of ISGs move out to the water phase, leading to the precipitation of IBU around the interface. The optimization of drug release profiles ensures sustained local drug release over seven days, with a burst release observed on the first day. Interestingly, different organic solvents show varying abilities to control DH release, with dimethyl sulfoxide (DMSO) demonstrating superior control compared to *N*-Methyl-2-pyrrolidone (NMP). MD simulations using AMBER20 software provide valuable insights into the movement of individual molecules, as evidenced by root-mean-square deviation (RMSD) values. The addition of IBU to the system results in the retardation of IBU molecule movement, particularly evident in the DMSO series, with the diffusion constant value of DH reducing from 1.2452 to 0.3372 and in the NMP series from 0.3703 to 0.2245 after adding IBU. The RMSD values indicate a reduction in molecule fluctuation of DH, especially in the DMSO system, where it decreases from over 140 to 40 Å. Moreover, their radius of gyration is influenced by IBU, with the DMSO system showing lower values, suggesting an increase in molecular compactness. Notably, the DH-IBU configuration exhibits stable pairing through H-bonding, with a higher amount of H-bonding observed in the DMSO system, which is correlated with the drug retardation efficacy. These significant findings pave the way for the development of phase transformation mechanistic studies and offer new avenues for future design and optimization formulation in the ISG drug delivery systems field.

## 1. Introduction

The in-situ gel (ISG) system is a versatile drug delivery approach that initially exists as a liquid dosage form. It later undergoes a transformation into semisolid gels at the target site, facilitating sustained drug release and localized drug levels [1]. This liquid-to-gel conversion allows for more comfortable administration than solid or semi-solid implants. The solvent exchange-induced ISG represents an injectable drug delivery system that dissolves active ingredients in biocompatible solvents. Upon administration into an aqueous environment, the solution transforms into a gel-like state due to the influx and outflux migration of anti-solvent (aqueous fluid) and solvent, respectively.

Consequently, the entrapped drugs are gradually released from the precipitated matrix over time. This controlled release mechanism enhances localized drug delivery, minimizing systemic toxicity. The development of solvent-exchange-ISG starts with a polymer-based system and advances to incorporate various hydrophobic materials. Among them are phospholipids, cholesterol, and fatty acids, recently utilized as structure-forming components [2]. These materials bring distinct advantages to the ISG character, setting it apart from traditional ISG systems.

Ibuprofen (IBU), a propionic acid substance, is a hydrophobic material with a Log Ko/w value of 3.97. It is a widely used nonsteroidal anti-inflammatory drug (NSAID) for managing mild to moderate pain and inflammation, including dental pain [3]. Additionally, IBU has been recognized for its effectiveness in reducing the signs and severity of gingivitis [4,5,6]. Thus, the IBU compound is regarded as safe for the treatment of oral cavity inflammation [7]. Despite its apparent low aqueous solubility, IBU can be utilized as a gel-forming component in the ISG system to carry doxycycline hyclate (DH) and leverage its self-anti-inflammatory effect. This integration aims to improve the efficacy of the treatment. Previous research has emphasized studying the physicochemical properties, drug control performance, and biological properties concerning the effects of various solvents and IBU concentrations [8]. The findings highlighted the influence of solvent type and IBU concentrations on the performance of these properties. In this work, our objective is to gain molecular insights into the early stages of phase transformation behavior using computational molecular dynamic (MD) simulation tools and correlate them with macroscopic physicochemical properties.

Molecular dynamic (MD) simulation is a powerful technique for predicting the structural and functional properties of molecules, extracting significant information from dynamic systems. While ab initio quantum chemical methods involve considering electrons and protons through quantum equations [9,10], molecular mechanics procedures based on force fields focus solely on the energies of systems by considering the positions of nuclei. These methods provide a cost-effective and straightforward approach with satisfactory accuracy for systems involving many particles, such as biological materials in drug delivery systems [11]. In drug delivery systems (DDS), numerous MD simulations have been employed to explore physicochemical aspects at the molecular level, including molecular structures and intermolecular interactions [11,12,13,14]. Computational MD simulations offer a valuable tool to investigate drug delivery mechanisms at the molecular level. They serve as an alternative to expensive and time-consuming experimental procedures, allowing for the estimation of optimized conditions for the designed systems [15,16,17,18,19]. These simulations offer valuable insights into the nature of diverse systems formed by high-energy interactions, and their results are consistent with experimental data [20,21]. Consequently, MD simulations serve as an initial screening approach to evaluate various scenarios and reduce experimental costs [22]. Focusing on recent MD simulations conducted on various drug carrier materials, such as polymers, composites, nanocomposites, graphene derivatives, carbon nanotubes, fullerenes, DNAs, peptides, proteins, nanoparticles, liposomes, and micelles, which have been widely employed in designing drug delivery systems [16,23,24,25,26].

In addition to our research aims, notable studies that have utilized MD simulations to gain insights into ISG drug delivery systems are worth mentioning. For instance, Chantadee T. et al. employed MD tools to elucidate the solvent exchange-induced crystallization behavior of various fatty acids and the controlled release ability of the system against vancomycin HCl. Their simulations successfully revealed the crystallization of different fatty acids and the mobility of vancomycin HCl outward from the fatty acid matrix to the aqueous phase [27]. Similarly, Lertsuphotvanit N. and colleagues performed conventional and MD simulation experiments to uncover the mechanism of borneol matrix formation in an anti-solvent-induced borneol-based in-situ matrix system [28].

**Figure 1:**
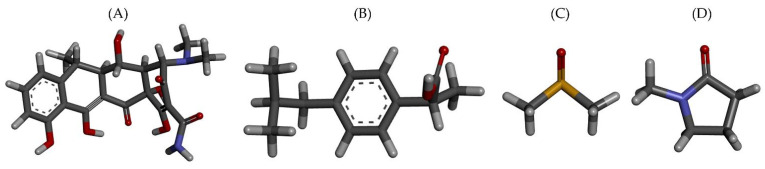
3-D structure of (**A**) DH; (**B**) IBU; (**C**) DMSO, and (**D**) NMP (gray, white, red, blue, and yellow sticks represent C, H, O, N and S atom, respectively).

This research aims to bridge the gap between experimental investigations and computational simulations. IBU was chosen as the gel former material of a DH-loaded ISG, wherein IBU was dissolved in different organic solvents. The molecular structures of DH, IBU, dimethyl sulfoxide (DMSO), and *n*-methyl pyrrolidone (NMP) are illustrated in Figure 1A–D. Utilizing over 200 ns of MD simulations on several models, as presented in Table 1, we aim to gain further insights into the early stages of the in-situ formation process, the molecular flow of components, and the interactions between the drug and the IBU gel. These factors significantly influence the system’s performance, including releasing behavior, self-gel formation ability, and sustained drug release capabilities. The study’s outcomes provide a platform for future advancements in the field of ISG DDSs and pave the way for the development of innovative approaches.

## 2. Materials and Methods

### 2.1. Materials

IBU (Lot No. 4000/1101/18/A-0150B, P.C. Drug Center Co., Ltd., Bangkok, Thailand) was used as the matrix-forming agent. DH (Lot No. 20071121, Huashu Pharmaceutical Corporation, Shijiazhuang, China) was used as the antimicrobial drug. NMP (≥99.5%, Lot No. 144560-118, QreC, Auckland, New Zealand) and DMSO (≥99.9%, Lot No. 1862992, Fisher Chemical, Horsham and Loughborough, UK) were used as the solvents. Potassium dihydrogen orthophosphate (Lot No. E23W60) and sodium hydroxide (Lot No. AF310204) from Ajax Finechem, New South Wales, Australia, were used as the components of phosphate-buffered saline (PBS pH 6.8). Agarose (Lot No. H7014714, Vivantis, Selangor Darul Ehsan, Malaysia) was used to determine the gel formation behavior.

### 2.2. Preparation of the ISG

IBU-based ISG systems were prepared by dissolving various concentrations of IBU (10%, 20%, 30%, and 40% *w*/*w*) in DMSO and NMP. DH concentration of 5% *w*/*w* was added to IBU (40% *w*/*w*) preparation. The mixtures were prepared by a magnetic stirrer at room temperature until completely dissolved. The components of the formulations are shown in Table 1.

### 2.3. Surface Tension

The surface tension of each formulation was evaluated using a goniometer (FTA 1000, First Ten Angstroms, Newark, CA, USA). The measurement involved observing the change in the shape of a pendant drop of the formulation suspended in the air after injection. The goniometer was equipped with a pump, and the drop was injected at a controlled rate of 1.9 μL/s. The experiment was conducted in triplicate (*n* = 3) to ensure the accuracy and reliability of the surface tension measurements.

### 2.4. Water Tolerance Measurement

Water tolerance was assessed to determine the phase transition tolerability of the ISG system to maintain its clear solution state in the presence of an added aqueous phase without precipitating IBU. To conduct this test, 2.5 g of the ISG formulation was placed in a test tube. Subsequently, 20 µL of deionized water was carefully added using a micropipette. The mixture was then subjected to vortex mixing until turbidity occurred at both 25 °C and 37 °C. The calculation of water tolerance was carried out using Equation (1), and the experiments were performed in triplicate (*n* = 3).

(1) $$\%\mathrm{water}\;\mathrm{tolerance}=\frac{\mathrm{water}\;\mathrm{amount}\left(g\right)}{\mathrm{sample}\;\mathrm{amount}\;\left(g\right)+\mathrm{water}\;\mathrm{amount}\;\left(g\right)}\times 100\%$$

### 2.5. Gel Formation Study

In this study, the investigation of the ISG transformation was carried out by injecting the ISG using a 1-mL syringe and an 18-gauge needle into PBS (pH 6.8). The transformation process was monitored by capturing images at various time intervals (1, 5, 10, 15, and 30 min) after the addition of the aqueous phase of surrounding agarose, which induced phase separation of the formula and led to the gradual solution-gel transformation, as evidenced by the appearance of a turbid layer. Additionally, to further explore the microscopic aspect of the interface among ISGs, an aqueous phase was observed by cutting the agarose gel on a glass slide to the edge and dropping 50 µL of ISG onto it. The interface interaction was visualized using an inverted fluorescent microscope (TE-2000U, Nikon, Kaw, Japan), and images were captured at 1, 5, and 15 min to study the dynamics of the gel formation process at the interface.

### 2.6. In Vitro Drug Release Studies

The in vitro drug release behavior of the DH-loaded IBU-based ISG formulations and control formulations (DH dissolved in DMSO and NMP) was investigated using the porcelain cup (membrane-less method), which provides a fixed surface with no membrane. This method uses a small porcelain cup to hold the sample being studied. It creates conditions similar to how drugs are put into crevices or other specific sites. A total of 0.3 g of each sample was placed in the cup, followed by adding 80 mL of PBS (pH 6.8) in a glass bottle. The setup was then placed in an incubator at 37 °C and shaken at 50 rpm. At specific time points, 3 mL of the release medium was sampled, and an equal volume of fresh PBS was added to maintain a sink condition.

The DH and IBU concentrations in the release medium were determined at each time point using high-performance liquid chromatography (HPLC) with a C18 column and a mobile phase of 0.1% formic acid/acetonitrile/methanol. The gradient elution method was employed with a 1 mL/min flow rate. Calibration curves were generated using intact DH and IBU standards dissolved in the release medium.

### 2.7. Molecular Dynamics Simulation for Phase Transformation Study

The computer dynamics modeling technique was employed to gain insights into the phase inversion and molecular movement of each component within the ISGs formula when exposed to an aqueous environment. Atom coordinates for each substance were obtained from databases such as PubChem and Cambridge Crystallographic Data Centre (CCDC). For substances unavailable in the database, molecular orbital calculations using Gaussian09 (Gaussian, Inc., Wallingford, CT, USA) were performed to determine the parameters required for MD simulations. Gaussview6 was utilized to generate the data used by Gaussian09. Simulation models were established based on the molar ratios of each formulation’s components, comprising DH, IBU, and the organic solvent (DMSO or NMP), as depicted in Table 1.

The MD simulations were conducted using the Amber 20 software package (University of California, San Francisco, CA, USA), and the force field parameters for the molecules were generated utilizing the Antechamber module with the general AMBER force field [29]. The system, consisting of DH, IBU, and organic solvents, was placed within a periodic boundary box and solvated with TIP3P water. Subsequent energy minimization and heating to 310 K were performed using the sander module, followed by the MD simulation at 310 K using the pmemd module.

Simulation analysis involved the use of Visual Molecular Dynamics (VMD) ver. 1.9.4 software (The Theoretical and Computational Biophysics group, The Beckman Institute, University of Illinois at Urbana-Champaign, IL, USA) to calculate hydrogen bond (H-bond) occupancy and density of molecules. H-bond formation was determined based on criteria where the distance between the acceptor and donor atoms was less than 3.5 Å, and the angle formed by the acceptor, donor, and hydrogen atoms was less than 60°. Density profiles of DH and IBU molecules were probed using VMD’s density profile tool to observe the density distribution of molecules in the simulation box coordinates [30]. The root-mean-squared deviation (RMSD) of the matrix, IBU, and DH was obtained using the cpptraj module [29]. Diffusion constants, reflecting the migration distance from the initial position, were calculated using the ptraj module with the time-evolved trajectory of the simulation. The diffusion constants provided valuable information about the outflux/influx ability of DH, IBU, organic solvents, and water [29].

For visual clarity and to determine the configuration of IBU and DH, the positions of certain molecules were transferred to the x-, y-, or z-direction based on the length of the periodic boundary box in the visualization of simulation structures. The drawing of simulation structures omitted irrelevant molecules to ensure clarity and was visualized using Chimera ver. 1.13.1rc (Resource for Biocomputing, Visualization, and Informatics University of California, San Francisco, CA, USA) and VMD programs, focusing on intermolecular interactions between IBU and DH.

### 2.8. Statistical Analysis

All data were examined using the one-way analysis of variance ANOVA followed by the LSD post-hoc test. The analysis was conducted using SPSS for Windows (version 11.5). The significant level was set at *p* < 0.05.

## 3. Results and Discussion

### 3.1. Water Tolerance

**Figure 2:**
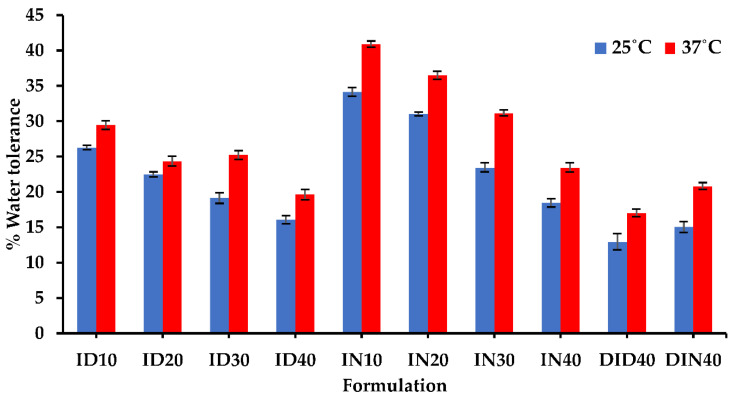
% water tolerance value of IBU-based ISG formulations.

In this research, water was utilized as a trigger to induce the transformation of the drug delivery system from a solution to a gel state through a solvent removal mechanism. Hence, a crucial parameter to assess the system’s ability to withstand exposure to aqueous environments is the water tolerance value. This value determines the minimum amount of aqueous phase required to induce complete phase separation in solvent removal-induced in-situ gel (ISG) systems. The water tolerance, which indicates the amount of water needed to achieve turbidity (endpoint), is an essential factor in determining the system’s water tolerance [2]. The water tolerance trend is presented in Figure 2. The water tolerance exhibited a noticeable decrease, indicating a higher sensitivity to the aqueous phase. As the concentration of IBU increased, the solvent became insufficient to dissolve the drug, resulting in the diffusion of the solvent out of the system. Furthermore, adding DH to the formulation contributed to a decrease in water tolerance. The increase in IBU concentration and the addition of DH led to an increase in the total solute molecules and a decrease in the solvent component of the system. Consequently, IBU reached its saturation point more easily due to its low hydrophilicity. However, an interesting finding emerged when exploring the impact of temperature on water tolerance. Increasing the surrounding temperature from room to body temperature showed a significant enhancement in water tolerance for all formulations. The water tolerance value of the entire series exhibited an increasing trend with the temperature rise. This observation suggests that higher temperatures promote improved solubility and dispersion of the components within the system, resulting in a higher tolerance to the aqueous phase. The enhanced water tolerance at elevated temperatures could be attributed to increased molecular mobility and improved drug dissolution. It is worth noting that the effect of temperature on water tolerance should be considered in the design and optimization of the drug delivery system. By leveraging the temperature-dependent behavior, it may be possible to fine-tune the formulation to achieve the desired drug release characteristics under specific physiological conditions. In summary, while the addition of DH and the increase in IBU concentration led to a decrease in water tolerance, the influence of temperature proved to be a crucial factor. The observed trend of increasing water tolerance with higher surrounding temperatures highlights the significance of temperature control in optimizing the drug delivery system, and their water tolerance profiles were used to design the molecular dynamic simulation system.

### 3.2. Surface Tension

Surface tension is a fundamental property that characterizes the behavior of liquid surfaces and plays a crucial role in various phenomena, including wetting, spreading, and drug delivery [31]. DMSO and NMP, the organic solvents used in this study, are polar aprotic solvents known for their strong dipole forces and hydrogen bonding interactions between their molecules. On the other hand, IBU and DH, the components introduced into the system, exhibit a mono-carboxylic acid and tetracycline structure, respectively, which can contribute to steric hindrance effects. The addition of IBU to the organic solvents resulted in a decrease in the surface tension of the formula (Table 2). Furthermore, the surface tension experienced a further decrease following the introduction of DH. This reduction in surface tension can be attributed to the interference of dissolved IBU and DH molecules, disrupting the attractive or cohesive forces among solvent molecules in the surface region [32]. The altered molecular interactions at the surface of the formulation, influenced by the presence of IBU and DH, lead to a decrease in surface tension. This finding has potential implications for the spreading and wetting properties of the drug delivery system, influencing its interaction with biological surfaces during administration.

### 3.3. Self-Gel Transformation Phenomenon of ISGs

**Figure 3:**
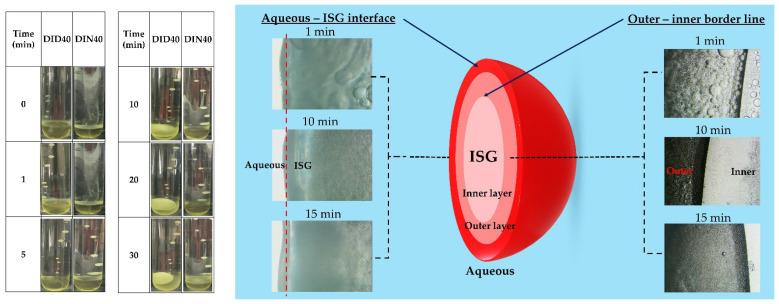
Phase transformation of IBU-based ISGs at several time points in PBS pH 6.8 at macroscopic level and microscopic level via inverted microscope.

To investigate the self-gel transformation behaviors of these formulations, a combination of macroscopic and microscopic studies was conducted, as shown in Figure 3. In the macroscopic research, the formulation was injected into a phosphate buffer solution (PBS) at pH 6.8 to simulate the inter-crevicular fluid in the periodontal pocket of periodontitis patients. The transformation process was observed over 30 minutes. The DID40 formulation gradually transitioned from a yellowish clear liquid to a turbid yellow gel with spherical shapes. Additionally, a clear yellow liquid layer separated from the PBS and accumulated at the bottom of the test tube. The DIN40 formulation exhibited slower transformation than DID40. It presented a similar appearance change at 30 min as the DID40 formulation did at 5 min. In the microscopic study, an inverted microscope was employed to examine the DID40 formulation against an agarose gel at pH 6.8. The microscopic analysis revealed a fascinating two-layer phenomenon. The outer layer consisted of droplets in motion, moving outward from the interlayer towards the aqueous phase at the interface and accumulating at the interlayer between the outer and inner layers. The inner layer remained unchanged and appeared as a clear liquid. Over time, the droplets progressively decreased in size, resulting in increased turbidity and decreased velocity. After 10 min, thin crystal layers of IBU began to form at the interface, and as time passed, the crystal layer became thicker. Moreover, in previous work, it was reported that the velocity and fluctuation of the droplet depend on the IBU content and solvent type. It was demonstrated that an increase in IBU concentration led to a rise in the fluctuation of the droplet, and the droplet in DMSO exhibited more excited movement than in NMP [8]. The macroscopic findings demonstrate the visual changes and separation phenomena occurring within the formulation, while the microscopic study reveals the behavior of droplets within the gel matrix. The observed movement of droplets in the microscopic study can be explained by the principles of phase inversion, which are driven by thermodynamic considerations and the reduction of the free energy of the system. When water-miscible solvents are present in the formulation, the diffusion of water into the ISG solution leads to a decrease in solvent concentration and the formation of unstable compositions. This triggers a phase separation process, resulting in the formation of two distinct phases: an outer layer consisting of droplets of the IBU-lean phase and an IBU-rich phase with a demixing gap, while the inner layer remains a single homogeneous phase. The movement of droplets towards the aqueous phase at the interface is driven by the affinity between the water-miscible solvent and water, as the system seeks to minimize the free energy and achieve a more stable state. This leads to the rapid migration of tiny droplets from the interlayer to the aqueous phase, where the water concentration is higher. The slower movement and accumulation of larger droplets at the interlayer can be attributed to differences in droplet size, viscosity, and interfacial interactions [33]. These factors influence the dynamics of droplet movement during the self-gel transformation process. Furthermore, the accumulation of IBU crystals at the interface can be explained by the preferential solubility of IBU in the aqueous phase compared to the polymer-rich phase. As water penetrates the interlayer, it carries dissolved IBU, and upon reaching the interface, the solubility of IBU decreases, leading to the precipitation and accumulation of IBU crystals. Understanding the thermodynamic principles underlying phase inversion, the formation of polymer-lean droplets and polymer-rich phases, and the interplay between droplet characteristics and interfacial phenomena [34]. Most of the phase transformations observed in previous studies on polymer or gel-forming materials in in-situ gel systems (ISGs) or in-situ forming matrices (ISMs) (e.g., PLGA, ethyl cellulose, shellac, fatty acid, cholesterol, borneol, Eudragit RS) resulted in the sudden formation of a solid matrix upon injection into the target site, making it challenging to study the phase transformation process [1,2,34]. However, IBU transforms into a gel form, allowing continuous observation of the transformation process. This unique property of IBU-based in-situ gels offers valuable insights into the phenomenon during transformation, contributing to a better understanding of the underlying mechanisms.

### 3.4. In Vitro Drug Release

**Figure 4:**
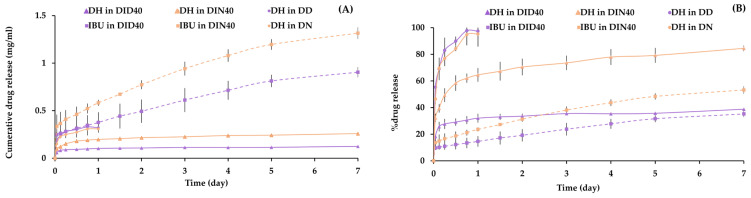
Drug release pattern of cumulative drug release in concentration (mg/mL) (**A**) and % drug release unit (**B**).

The drug release study of DH-loaded IBU-based in-situ gels (ISGs) revealed important findings regarding the release behavior of the drugs over time. The cumulative drug release concentrations of DH and IBU were measured at different time points to assess the sustained release capabilities of the ISGs, as shown in Figure 4A in the concentration of cumulative drug release (mg/mL) and Figure 4B in % drug release. The control groups, such as DD and DN, were released suddenly into a PBS pH 6.8 solution. DD and DN exhibited burst release to 100% within the 1st day. However, DID40 and DIN40 exhibited burst release at the initial stage or within 3 and 18 h, respectively. After the initial stage, the DH release rate decreased and turned into a controlled release over time. This behavior can be attributed to the state of the gels, where the solid mass of the hydrophobic IBU matrix may obstruct DH release more than the liquid and gel states, contributing to the controlled release behavior.

On the 1st day, DH’s cumulative drug release concentration was 0.32 mg/mL for the DID40 formulation and 0.31 mg/mL for the DIN40 formulation. The cumulative drug release concentration for IBU was 0.376 mg/mL for DID40 and 0.583 mg/mL for DIN40. These results demonstrate the initial burst release of both drugs from the ISGs. Upon further analysis, it was observed that the release rates of DH and IBU decreased over time, indicating controlled release characteristics. On the 7th day, DH’s cumulative drug release concentration increased to 0.1253 mg/mL for DID40 and 0.259 mg/mL for DIN40. The cumulative drug release concentration for IBU reached 0.905 mg/mL for DID40 and 1.316 mg/mL for DIN40. These findings highlight the sustained release of DH and IBU from the ISGs, maintaining therapeutic concentrations over the study period. To evaluate the controllability of DH release, a comparison was made with Atridox^®^, a DH-containing product that uses poly(D, L-lactide) as a polymer. Atridox^®^ controls drug release for seven days after cleaning the periodontal pocket with an irrigating solution [1,35]. In the case of DID40 and DIN40 ISG formulations, the percentage of DH release on the seventh day was 38.72 ± 0.19% and 84.49 ± 2.08%, respectively. These ISGs exhibited efficient controlled DH release, ensuring sustained local drug release in the periodontal pocket and enhancing patient compliance by reducing medication frequency.

In summary, The ISGs exhibited an initial burst release of both drugs, followed by sustained and controlled release throughout the study period. The cumulative drug release concentrations demonstrated the sustained release capabilities of the ISGs, indicating their potential for therapeutic applications. The controlled release characteristics were influenced by the state of the gels and the hydrophobic IBU matrix. Moreover, comparisons with existing products underscored the significance of achieving controlled and sustained drug release in periodontal treatment. These findings contribute to a better understanding and optimization of drug release profiles, facilitating enhanced patient compliance and improved treatment outcomes.

### 3.5. In Silico Results of MD Simulation

**Figure 5:**
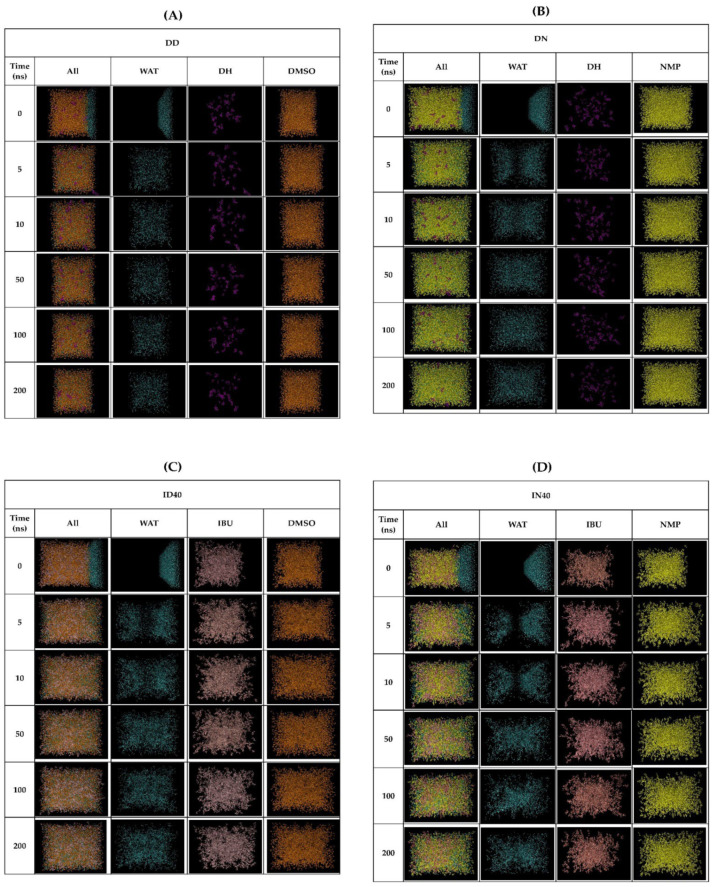

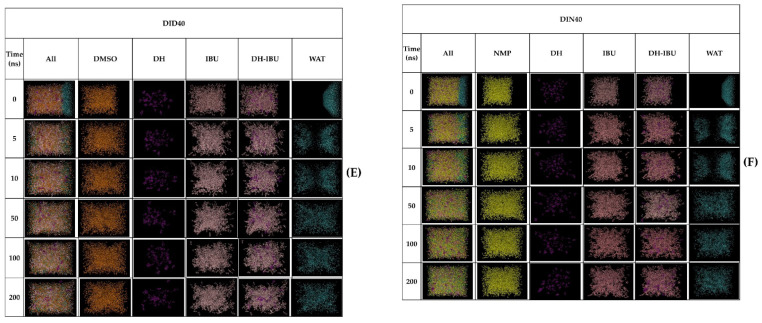
Molecular dynamic modeling of solvent exchange mechanism focuses on the interface of each ISGs against aqueous, (DD (**A**), DN (**B**), ID40 (**C**), IN40 (**D**), DID40 (**E**) and DIN40 (**F**) after running in MD simulation in 200 ns.

**Figure 6:**
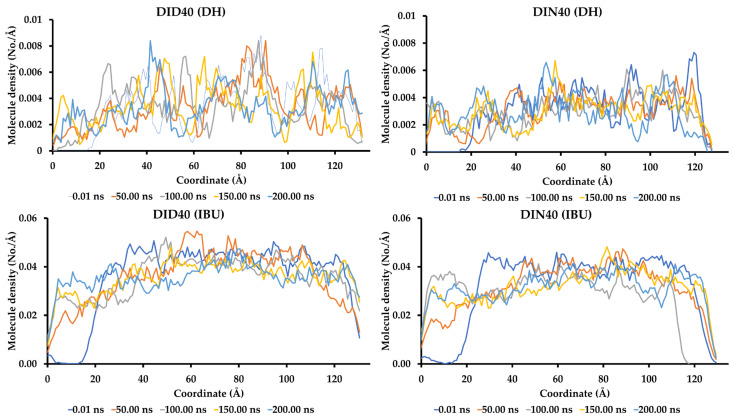

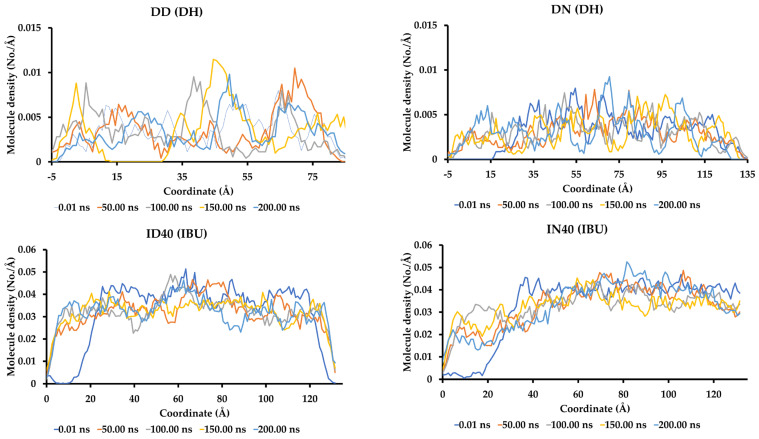
The density profile of IBU and DH molecules in the coordinate of different formulation boxes after contact with the water box.

In the MD simulation experiment, we designed an experimental setup to mimic the interface region between various IBU-based ISG phases (left side box) and the aqueous phase (right side box). The molecular ratio was determined based on the formula content and water tolerance value to determine the minimum water content around the interface that induces phase transformation behavior, as depicted in Figure 5. In Figure 5A,B, the DD and DN formulations exhibited freely moving DH molecules without significant agglomeration. However, the movement of water (WAT) molecules showed different behavior, with faster inward diffusion into the DD system than the DN system. This was observed through the time interval of WAT diffusion until homogeneity was achieved in the box. Simulation animations of the ID40 and IN40 formulations are shown in Figure 5C,D, respectively. The simulations revealed no difference between the two IBU formulations. Over time, IBU gradually formed bulk structures while exhibiting continuous movement within their units for up to 200 ns. This indicated that IBU did not undergo solid precipitation during the initial stage of phase transformation but rather formed clusters of IBU molecules. In terms of WAT molecule diffusion, the ID40 system showed faster inward diffusion into the ISGs box compared to the IN40 system, similar to the observations in the DD and DN simulations. For the DID40 and DIN40 systems, as Figure 5E,F, WAT inflation occurred at a slower rate compared to ID40, IN40, DD, and DN. Moreover, the DMSO series showed faster WAT movement than the NMP series. The agglomeration of IBU molecules in IBU-based ISGs systems was confirmed by the lack of change in the density pattern of molecules within the 40–80 Å coordinate range after 100 ns, as shown in Figure 6. On the other hand, DH exhibited fluctuating density movement patterns in both formulations with and without IBU, as depicted in Figure 5C,D.

**Figure 7:**
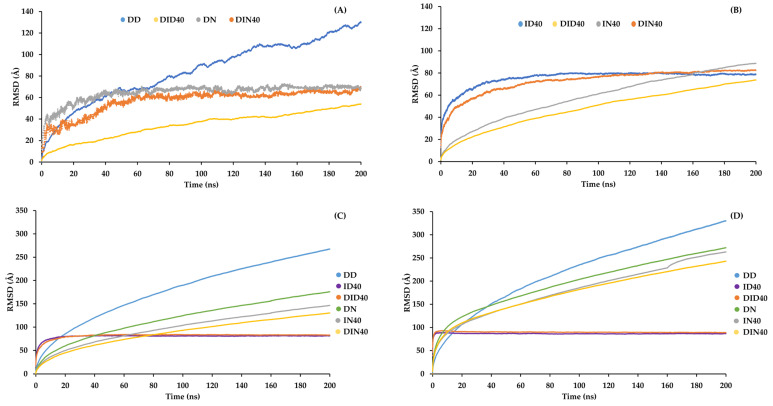
Root mean square deviation (RMSD) of (**A**) DH, (**B**) IBU, (**C**) organic solvent and (**D**) water conformation relative to the starting point of the MD simulations box contact with water.

The RMSD parameter reflects the fluctuations of molecules during the simulation. Figure 7 illustrates the RMSD values for each molecular type over time. All graphs exhibit a similar pattern, with an initial phase of high RMSD values followed by a plateau phase where the RMSD values remain constant or increase slightly. Although DH molecules showed unrestricted movement without being fully obstructed by other molecules in the system, the RMSD values of DH in the DMSO system were higher than those in the NMP system. Specifically, the addition of IBU dramatically reduced the RMSD values of DH in both the first and second phases of the DMSO system.

In contrast, the NMP series only showed a decrease in the RMSD values of DH during the first phase but no significant difference during the second phase. This indicates that DH movement was effectively restrained by IBU, especially in the DID40 system. The effect of IBU on DH retardation is further supported by analyzing the RMSD values of other molecules. The RMSD values of IBU (Figure 7B) were slightly lower in the NMP series than in the DMSO series, but both systems showed similar values during the second phase. IBU in the DMSO system exhibited lower mobility than the NMP system or showed a higher tendency to pack together more efficiently. The RMSD patterns of organic solvents and WAT molecules (Figure 7C,D) were similar in all the formulations. In the DMSO series, the RMSD values demonstrated significantly lower values in the IBU-based ISGs system than those without IBU. In the NMP series, there was a slight decrease in RMSD values after the addition of IBU. The RMSD analysis of aqueous and organic solvent results provides insights into the packing of IBU through aqueous and organic solvent exchanges. Specifically, the DID40 DMSO system and the water molecules showed higher mobility, indicating lower mobility or stronger packing of IBU compared to the DIN40 system and further contributing to the retardation of DH movement compared to IBU in the DIN40 system. To further investigate the molecular movement and diffusion throughout the phase transformation, diffusion constant values were calculated and are presented in Table 3. The diffusion constants provide information related to the RMSD results. A comparison between the DMSO and NMP systems revealed that the diffusion constants of DH, organic solvents, and WAT in the DMSO system were significantly reduced upon the addition of IBU compared to the NMP system.

**Figure 8:**
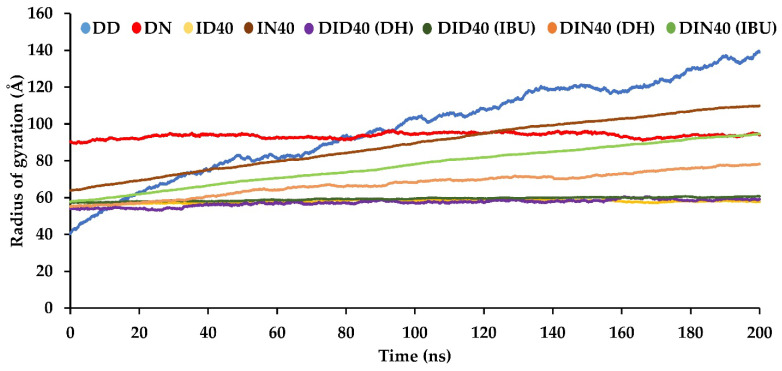
The radius of gyration of DH and IBU conformation of the MD simulations box contact with water.

The radius of gyration measures the size and compactness of molecules. It quantifies the distance of atoms or residues from the molecule’s center of mass. A smaller radius of gyration indicates a more compact or extended structure, while a larger radius indicates the opposite [36]. In our study, the Amber program was used to calculate the radius of gyration for atoms or residues in the molecules to provide structural and dynamic information. The size of DH was found to decrease upon the addition of IBU, as seen in Figure 8. Furthermore, the DMSO series exhibited denser compaction of both DH and IBU compared to the NMP series, indicating that the presence of IBU influenced the retardation of DH distribution within the box, and the type of organic solvent had an impact on the rate of IBU formation. Hydrogen bonds are crucial in intermolecular chemical bonding and can significantly influence physicochemical processes.

**Figure 9:**
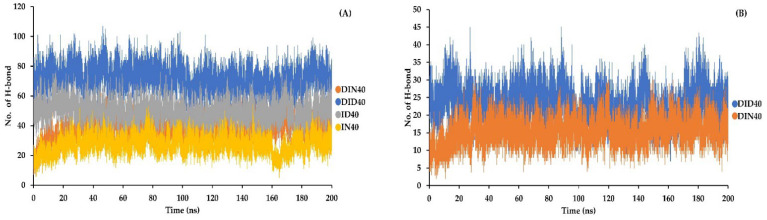
Change in number of hydrogen bonds of (**A**) IBU-IBU and (**B**) DH-IBU molecule of formulations box contact with water.

The hydrogen bond amounts were determined using the VMD software based on geometric criteria, including a distance criterion within 3.5 Å between chosen donor-acceptor pairs and an angle criterion of less than 60° [37,38]. The number of hydrogen bonds formed between IBU-IBU and DH-IBU was investigated, as shown in Figure 9A,B, respectively. The IBU-IBU interaction was the highest in the DID40 system, followed by the DIN40 system, ID40 system, and IN40 system. Regarding the DH-IBU interaction, the DID40 system exhibited a higher number of hydrogen bonds compared to the DIN40 system, which is consistent with the RMSD and radius of gyration results, confirming the significant interaction between the IBU structure and DH. Hydrogen bonding was crucial for the DH movement facilitated by IBU, as supported by the aforementioned evidence.

**Figure 10:**
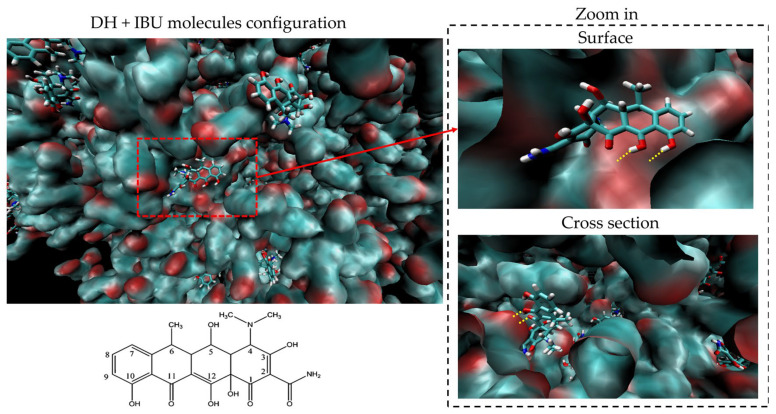
Configuration between DH and IBU at the final stage of MD simulation in DID40 systems. (DH and IBU molecules were demonstrated as stick and rough surface mass structures, respectively. Blue, white, red, and dark blue structures or surfaces are represented C, H, O and N atoms, respectively. The yellow bonds are intermolecular H-bonding of DH and IBU). A 2D-chemical structure represents the C atom numbering of the DH molecule.

The final configuration of the DH-IBU complex in the DID40 system is presented in Figure 10. To facilitate visualization, IBU and DH are depicted as a singular matrix and stick structure, respectively, with different colors representing each element. The configuration of the complex exhibits various formation types, with the majority of them showing interesting intermolecular interactions such as hydrogen bonding (indicated by yellow dotted lines) between the carboxyl group of IBU (red surface of the matrix) and the hydroxyl groups at C3, C10, and C12 of DH. This configuration represents one of the most stable forms of the DH-IBU complex. That might clarify the H-bonding location between DH and IBU that is influenced to retardation of DH penetration and release out the IBU-based ISGs system to the environment.

In summary, the MD simulation provided valuable insights into the behavior of IBU-based ISGs at the interface with the aqueous phase. The simulations demonstrated phase transformation behavior, diffusion patterns of molecules, changes in RMSD values, compactness of molecules, hydrogen bonding interactions, and the configuration of the DH-IBU complex. These findings contribute to a better understanding of the molecular dynamics and interactions within IBU-based ISGs, aiding in the development of controlled drug release systems.

## 4. Conclusions

This research work found the influence of DH and IBU is reducing the surface tension of the IBU-based ISGs system via steric hindrance effect and reducing the water tolerance of the system was reduced by decreasing organic solvent composition. The macroscopic presented a higher gel formation rate in the DMSO series that related with the water tolerance value and microscopic aspect of phase transformation insight presented phase transformation behaviour around interface as moving outward of IBU-solution droplet and accumulated of IBU droplet at aqueous-ISGs interface. The crystallization of IBU occurs when the concentration of IBU reaches the crystallization point of IBU via the solvent exchange effect. Furthermore, the investigation of drug release behaviour provided the release behavior of both DH and IBU from ISGs was determined to be sustained over seven days with burst releasing of DH on 1st day. Interestingly, different organic solvents show varying abilities to control DH release, with DID40 presenting higher controllability of DH release than the DIN40 formula. For the molecular aspect, MD simulations are used to mimic phase transformation behaviour at the interface region between the ISG phases and the aqueous phase in the supremely initial stage. IBU molecules presented gradually forming compaction of structures over time. Their formation behaviour was influenced by organic solvent type; IBU molecules in the DMSO series presented more compactness with higher H-bonding of IBU itself than in the NMP series. DH and solvent movement were reduced after IBU adding. Moreover, the compactness of the structures, with the reduction of DH distribution, is influenced by the addition of IBU.

Additionally, the analysis of hydrogen bonding revealed important intermolecular interactions, particularly between IBU and DH. These findings contribute to understanding the phase transformation process, drug release mechanisms, and molecular interactions within the ISGs. Such computational insights are crucial for explaining phase transformation with controlled drug release property of ISGs drug delivery systems for therapeutic applications.

## Figures and Tables

**Table 1:** Composition (A) and amount (B) of different molecules of DH-loaded IBU-based ISG formulations.

(**A**)						
**Formulation**	**IBU** **(% *w*/*w*)**		**DH** **(% *w*/*w*)**		**Organic Solvent** **(Adjust to 100% *w*/*w*)**	
DD	-		5		DMSO	
DN	-		5		NMP	
ID10	10		-		DMSO	
ID20	20		-		DMSO	
ID30	30		-		DMSO	
ID40	40		-		DMSO	
IN10	10		-		NMP	
IN20	20		-		NMP	
IN30	30		-		NMP	
IN40	40		-		NMP	
DID40	40		5		DMSO	
DIN40	40		5		NMP	
(**B**)						
**Molecular Dynamic Box Details**	**DD**	**DN**	**ID40**	**IN40**	**DID40**	**DIN40**
Amount of DH molecules	80	80	0	0	80	80
Amount of IBU molecules	0	0	1600	1600	1440	1440
Amount of DMS molecules	8640	0	6400	0	5040	0
Amount of NMP molecules	0	6880	0	4800	0	4000
Amount of WAT molecules	7896	7973	8837	10,029	7458	9074
Mole ratio of DH:IBU:(DMS/NMP):WAT	1:0:108:98.7	1:0:86:99.7	0:18:72:99.5	0:18:54:112.8	1:18:63:93.2	1:18:50:113.4
Total amount of molecules in the system	16,616	14,933	16,837	16,429	14,018	14,594
Total amount of atoms in the system	166,408	138,490	143,311	159,687	124,774	143,222

**Table 2:** Surface tension of organic solvents and doxycycline-loaded ibuprofen-based ISGs (*n* = 3).

Formula	Surface Tension (mN/m)
DMSO	43.95 ± 0.13
DD	41.72 ± 0.28
ID40	35.10 ± 0.87
DID40	34.81 ± 1.88
NMP	39.31 ± 0.28
DN	38.45 ± 0.11
IN40	36.91 ± 0.24
DIN40	36.73 ± 0.89

**Table 3:** Diffusion constant of each substance in formulations box contact with water.

Composition	Formulation					
	DD	DN	ID40	IN40	DID40	DIN40
DH	1.2452	0.3703	-	-	0.3372	0.2245
IBU	-	-	1.4820	0.6618	0.7130	0.4529
DMSO	5.8256	-	4.4566	-	2.6423	-
NMP	-	2.1479	-	2.0939	-	1.2914
WAT	9.2859	4.2385	8.1378	4.5864	5.6385	3.7678

## Data Availability

The data presented in this study are available at the request of the corresponding author.

## References

[1] Chuenbarn T., Chantadee T., Phaechamud T. (2022). Doxycycline hyclate-loaded Eudragit^®^ RS PO in situ-forming microparticles for periodontitis treatment. J. Drug Deliv. Sci. Technol..

[2] Chantadee T., Santimaleeworagun W., Phorom Y., Chuenbarn T., Phaechamud T. (2020). Vancomycin HCl-loaded lauric acid in situ-forming gel with phase inversion for periodontitis treatment. J. Drug Deliv. Sci. Technol..

[3] Irvine J., Afrose A., Islam N. (2018). Formulation and delivery strategies of ibuprofen: Challenges and opportunities. Drug Dev. Ind. Pharm..

[4] Su J., Gu J., Dong Z., Mei B. (2013). Ibuprofen rescues abnormalities in periodontal tissues in conditional presenilin 1 and presenilin 2 double knockout mice. Int. J. Mol. Sci..

[5] Agossa K., Delepierre A., Lizambard M., Delcourt-Debruyne E., Siepmann J., Siepmann F., Neut C. (2020). In-situ forming implants for dual controlled release of chlorhexidine and ibuprofen for periodontitis treatment: Microbiological and mechanical key properties. J. Drug Deliv. Sci. Technol..

[6] Maddiboyina B., Krishna A., Sri R., Kalyani B., Jabeena S., Sharmila S., Lakshmibai V. (2020). Preparation and evaluation of ibuprofen in-situ periodontal gel. Int. J. Allied Med. Sci. Clin. Res..

[7] Moura S.K.S.C.F., dos Santos M.L.V., do Nascimento L.A., da Silva M.F.A., de França G.M., da Costa L.M., Medeiros A.C., Araújo-Júnior R.F., de Araújo A.A., Oliveira C.N. (2022). Design of a thermosensitive ibuprofen-loaded nanogel as smart material applied as anti-inflammatory in tooth bleaching: An in vivo study. J. Drug Deliv. Sci. Technol..

[8] Puyathorn N., Senarat S., Lertsuphotvanit N., Phaechamud T.A.-O. (2023). Physicochemical and Bioactivity Characteristics of Doxycycline Hyclate-Loaded Solvent Removal-Induced Ibuprofen-Based In Situ Forming Gel. Gels.

[9] Shariatinia Z., Abdollahi-Moghadam M. (2018). DFT computations on surface physical adsorption of hydrocarbons produced in the Fischer-Tropsch synthesis on a CNT/Co nanocatalyst. J. Saudi Chem. Soc..

[10] Kazemi S., Daryani A.S., Abdouss M., Shariatinia Z. (2016). DFT computations on the hydrogen bonding interactions between methacrylic acid-trimethylolpropane trimethacrylate copolymers and letrozole as drug delivery systems. J. Theor. Comput. Chem..

[11] Li J., Chen C., Zhang J., Zhang L., Liang L., Kong Z., Jia-Wei S., Xu Y., Wang X., Zhang W. (2020). Molecular dynamics study on loading mechanism of chitosan into boron nitride nanotubes. J. Mol. Liq..

[12] Shariatinia Z., Mazloom-Jalali A. (2020). Molecular dynamics simulations on chitosan/graphene nanocomposites as anticancer drug delivery using systems. Chin. J. Phys..

[13] Jalali A.M., Shariatinia Z., Taromi F.A. (2017). Desulfurization efficiency of polydimethylsiloxane/silica nanoparticle nanocomposite membranes: MD simulations. Comput. Mater. Sci..

[14] Maleki R., Afrouzi H.H., Hosseini M., Toghraie D., Piranfar A., Rostami S. (2020). pH-sensitive loading/releasing of doxorubicin using single-walled carbon nanotube and multi-walled carbon nanotube: A molecular dynamics study. Comput. Methods Programs Biomed..

[15] Shariatinia Z., Azar A.T. (2021). Chapter 10—Molecular Dynamics Simulations on Drug Delivery Systems. Modeling and Control of Drug Delivery Systems.

[16] Mollazadeh S., Sahebkar A., Shahlaei M., Moradi S. (2021). Nano drug delivery systems: Molecular dynamic simulation. J. Mol. Liq..

[17] Asmari M., Wang X., Casado N., Piponski M., Kovalenko S., Logoyda L., Hanafi R., El Deeb S. (2021). Chiral Monolithic Silica-Based HPLC Columns for Enantiomeric Separation and Determination: Functionalization of Chiral Selector and Recognition of Selector-Selectand Interaction. Molecules.

[18] Berillo D.A.-O., Yeskendir A., Zharkinbekov Z.A.-O., Raziyeva K., Saparov A.A.-O. (2021). Peptide-Based Drug Delivery Systems. Medicina.

[19] Man V.A.-O., Li M.A.-O., Wang J., Derreumaux P., Nguyen P.A.-O.X. (2019). Interaction mechanism between the focused ultrasound and lipid membrane at the molecular level. J. Chem. Phys..

[20] Pai R.V., Monpara J.D., Vavia P.R. (2019). Exploring molecular dynamics simulation to predict binding with ocular mucin: An in silico approach for screening mucoadhesive materials for ocular retentive delivery systems. J. Control. Release.

[21] Khalkhali M., Mohammadinejad S., Khoeini F., Rostamizadeh K. (2019). Vesicle-like structure of lipid-based nanoparticles as drug delivery system revealed by molecular dynamics simulations. Int. J. Pharm..

[22] Shariatinia Z., Ahmadi-Ashtiani A. (2019). Corrosion inhibition efficiency of some phosphoramide derivatives: DFT computations and MD simulations. J. Mol. Liq..

[23] Mollazadeh S., Mackiewicz M., Yazdimamaghani M. (2021). Recent advances in the redox-responsive drug delivery nanoplatforms: A chemical structure and physical property perspective. Mater. Sci. Eng. C.

[24] Vatanparast M., Shariatinia Z. (2019). Hexagonal boron nitride nanosheet as novel drug delivery system for anticancer drugs: Insights from DFT calculations and molecular dynamics simulations. J. Mol. Graph. Model..

[25] Vatanparast M., Shariatinia Z. (2018). AlN and AlP doped graphene quantum dots as novel drug delivery systems for 5-fluorouracil drug: Theoretical studies. J. Fluor. Chem..

[26] Vatanparast M., Shariatinia Z. (2019). Revealing the role of different nitrogen functionalities in the drug delivery performance of graphene quantum dots: A combined density functional theory and molecular dynamics approach. J. Mater. Chem. B.

[27] Chantadee T., Sirirak J., Hoshino T., Phaechamud T. (2021). Augmentative molecular aspect for phase inversion of vancomycin hydrochloride-loaded fatty acid in situ forming matrices. Mater. Des..

[28] Lertsuphotvanit N., Sirirak J., Tamdee P., Tuntarawongsa S., Phaechamud T., Chantadee T. (2023). Ways to Assess and Regulate the Performance of a Bi-Mechanism-Induced Borneol-Based In Situ Forming Matrix. Pharmaceutics.

[29] Shahab M., Akter S., Sarkar M.M.H., Banu T.A., Goswami B., Chowdhury S.F., Naser S.R., Habib M.A., Shaikh A.A., Saki M. (2023). Computational design of medicinal compounds to inhibit RBD-hACE2 interaction in the Omicron variant: Unveiling a vulnerable target site. Inform. Med. Unlocked.

[30] Giorgino T. (2014). Computing 1-D atomic densities in macromolecular simulations: The density profile tool for VMD. Comput. Phys. Commun..

[31] Agrawal G., Negi Y.S., Pradhan S., Dash M., Samal S.K., Tanzi M.C., Farè S. (2017). 3—Wettability and contact angle of polymeric biomaterials. Characterization of Polymeric Biomaterials.

[32] Qazi M.J., Schlegel S.J., Backus E.H.G., Bonn M., Bonn D., Shahidzadeh N. (2020). Dynamic Surface Tension of Surfactants in the Presence of High Salt Concentrations. Langmuir.

[33] McClements D.J. (2012). Nanoemulsions versus microemulsions: Terminology, differences, and similarities. Soft Matter.

[34] Parent M., Nouvel C., Koerber M., Sapin A., Maincent P., Boudier A. (2013). PLGA in situ implants formed by phase inversion: Critical physicochemical parameters to modulate drug release. J. Control. Release.

[35] Javali M.A., Vandana K.L. (2012). A comparative evaluation of atrigel delivery system (10% doxycycline hyclate) Atridox with scaling and root planing and combination therapy in treatment of periodontitis: A clinical study. J. Indian. Soc. Periodontol..

[36] Lobanov M.Y., Bogatyreva N.S., Galzitskaya O.V. (2008). Radius of gyration as an indicator of protein structure compactness. Mol. Biol..

[37] Crossley-Lewis J., Dunn J., Buda C., Sunley G.J., Elena A.M., Todorov I.T., Yong C.W., Glowacki D.R., Mulholland A.J., Allan N.L. (2023). Interactive molecular dynamics in virtual reality for modelling materials and catalysts. J. Mol. Graph. Model..

[38] Vahed M., Neya S., Matsuzaki K., Hoshino T.A.-O. (2018). Analysis of Physicochemical Interaction of Aβ(40) with a GM1 Ganglioside-Containing Lipid Membrane. J. Phys. Chem. B.

